# Beta-Glucans from Fungi: Biological and Health-Promoting Potential in the COVID-19 Pandemic Era

**DOI:** 10.3390/nu13113960

**Published:** 2021-11-06

**Authors:** Iwona Mirończuk-Chodakowska, Karolina Kujawowicz, Anna Maria Witkowska

**Affiliations:** Department of Food Biotechnology, Faculty of Health Sciences, Medical University of Bialystok, Szpitalna 37, 15-295 Bialystok, Poland; karolina.kujawowicz@sd.umb.edu.pl (K.K.); anna.witkowska@umb.edu.pl (A.M.W.)

**Keywords:** β-glucans, macrofungi, mushrooms, food, SARS-CoV-2, COVID-19

## Abstract

Beta-glucans comprise a group of polysaccharides of natural origin found in bacteria, algae, and plants, e.g., cereal seeds, as well as microfungi and macrofungi (mushrooms), which are characterized by diverse structures and functions. They are known for their metabolic and immunomodulatory properties, including anticancer, antibacterial, and antiviral. Recent reports suggest a potential of beta-glucans in the prevention and treatment of COVID-19. In contrast to β-glucans from other sources, β-glucans from mushrooms are characterized by β-1,3-glucans with short β-1,6-side chains. This structure is recognized by receptors located on the surface of immune cells; thus, mushroom β-glucans have specific immunomodulatory properties and gained BRM (biological response modifier) status. Moreover, mushroom beta-glucans also owe their properties to the formation of triple helix conformation, which is one of the key factors influencing the bioactivity of mushroom beta-glucans. This review summarizes the latest findings on biological and health-promoting potential of mushroom beta-glucans for the treatment of civilization and viral diseases, with particular emphasis on COVID-19.

## 1. Introduction

Mushrooms (macrofungi) have been a subject of interest to people for centuries. Edible mushrooms have been appreciated not only for their taste, but also for medicinal properties. The oldest data indicating the use of fungi by humans come from archaeological excavations dating back to about 8000 years BC. In the 1990s, two species of fungi were found by the corpse of an “ice man”, namely, *Piptoporus betulinus* (Bull.) P. Karst, and the *Fomes fomentarius* (L.) Fr, which may have served “Ötzi”, living 5000 years BC, as dressings or, presumably, they could have been treated as a cure for stomach problems. Another valued species with medicinal properties was *Fomitopsis officinalis* (Vill.) Bres used by the ancient Greeks and Romans as a cure for many diseases, such as excessive sweating during tuberculosis, dizziness, respiratory diseases, digestive problems, and even cancer. The therapeutic properties of fungi were earliest and most often used in the Far East, i.e., in China and Japan. In Europe, mushrooms were more frequently valued for their taste. The oldest text reports on the therapeutic properties of fungi date back to around the 1st century BC. They mention the Japanese shiitake (*Lentinula edodes* (Berk.) Pegler, *Ganoderma lucidum* (M.A. Curtis) P. Karst. Other species with documented medicinal properties in historical reports are *Amanita muscaria* (L.) Lam., used in the past to treat rheumatism and to restore the function of the secretory glands, and the *Lycoperdon* sp., used as an antihemorrhagic agent [1].

Apart from their nutritional value [2], mushrooms are attributed with a wide range of health-promoting properties [3,4,5,6,7,8,9]. They exhibit antioxidant [3], hypotensive [4], hypocholesterolemic [5], and hypoglycemic [6] as well as anticancer [7], immunomodulating [7], antiviral [8], and bacteriostatic properties [9]. The health properties of fungi result from the presence of biologically active substances, including phenolic compounds and vitamins (A, E, C), antioxidant elements, and amino acids [10,11,12]. In recent years, the greatest interest has been focused on beta-glucans, especially on the possibility of their use in the treatment of civilization diseases [13] and COVID-19 [14].

The aim of this review was to summarize the latest findings on biological and health promoting properties of mushroom beta-glucans with a potential to support the treatment of many disorders, including civilization and viral diseases, with particular emphasis on COVID-19.

## 2. Review

### 2.1. Classification and Structure of Mushroom Glucans

The most common polysaccharides in fungi are chitin, hemicellulose, beta-glucans, alpha-glucans, mannans, xylans, and galactans [15,16].

Polysaccharides are a very diverse group of macromolecules whose monomers are linked by glycosidic bonds. Monomers in polysaccharides can be linked both linearly and form branched chains. The basic units forming the fungal polysaccharides may be glucose, fructose, glucuronic acid, arabinose, galactose, xylose, and mannose. Additionally, polysaccharides can be combined with peptides and proteins [17,18].

Fungal glucans can be linear or branched. The molecules of particular monosaccharides, mainly glucose, are connected by α- or β-glycosidic and also by the various types of different glycosidic linkages present in the same molecule. Mushroom D-glucans can have different linkage types, branching degrees, molecular weights, and solubility profiles [19,20].

**Figure 1:**
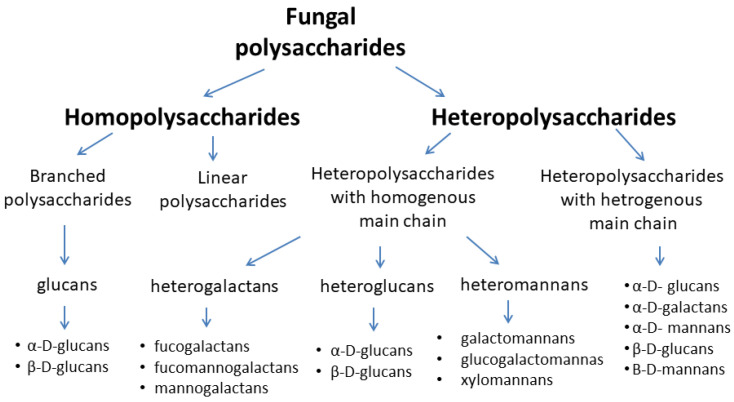
Classification of fungal polysaccharides [17,23,24].

Heteropolysaccharides, among them, heteropolysaccharides with a homogeneous main chain (heterogalactans, heteroglucans, and heteromannans) and heteropolysaccharides with a heterogeneous main chain, are a more diverse group of biologically active polysaccharides [17]. Due to the structure of the basic chain of these compounds, they are divided into mannans, xylans, galactans, and fructans. The side chains of these macromolecules may include arabinose, fructose, mannose, galactose, or glucose [17,19,21,22,23,24]. Figure 1 shows the classification of fungal main polysaccharides.

#### 2.1.1. General Characteristic of β-Glucans

Beta-glucans are polymers of β-D-glucose. They constitute part of cell walls of bacteria and plants, mainly algae and cereals, as well as microscopic fungi and macrofungi [25,26]. Beta-glucans have mainly a structure-forming role in the cell. They are divided into several different groups according to their structure, i.e., the degree of chain branching and the type of glycosidic bonds connecting the glucose monomers. β-1,3-glucans, without branches (linear), occur in bacteria. β-1,3-1,4-D-glucans are mainly found in cereal grains such as oats and barley, and marine algae such as brown algae [27], while β-1,3-1,6-D-glucans occur mainly in yeasts (β-1,3-glucans with long-branched chains β-1,6- (*Sacharomyces cerevisiae*)) and macrofungi (β-1,3-glucans with short-branched chains β-1,6-) [28].

Bioactivity of beta-glucans depends on the conformation of their molecules. Glucans, like other polymers, can adopt different chain conformations depending on the type of solvent, e.g., random coil, single helix, double helix, triple helix, worm-like, rod-like, and sphere-like shapes. However, beta-1,3–glucans with beta-1,6 branches or without branches make up a triple helix structure in its natural form and in aqueous solutions at room temperature. The triple helix structure determines the immunomodulatory and anticancer properties of beta-glucans [29].

β-glucans are essential components of cell walls in cereal grains, mainly oats, barley, rye, and millet. They occur in the outermost layer of grains (aleuronic layer) mainly as unbranched β-D-glucose chains linked by β-1-3 or β-1-4 glycosidic bonds. Their content, however, is quite low, ranging from 4–7%. The highest molecular weights among cereal grain glucans are observed in β-glucans of oats (3,000,000 Da) and barley (2,100,000 Da) [30,31].

Cereal β-glucans show a number of health properties. They lower blood cholesterol and glucose [32], support the treatment of obesity, inflammation of the intestine, and gastric mucosa, and take part in the microbiota modulation [5,32,33].

Beta-glucans are also a part of the inner layer of the yeast cell walls, where their content varies widely, ranging from 78% to 84% [33]. The content of glucans largely depends on the method of yeast cultivation. There are several types of glucans in the yeast cell wall, differing in the type of bonds and the branching of molecules. Among others, there are high-molecular-weight, insoluble 1-3-β-glucans with a few side branches connected to the main chain by a β-1-6 bond, low-molecular-weight, highly branched β-1-6-glucans with side branches connected to the main chain by a β-1-3 bond, and low-molecular-weight, soluble 1-3-β-glucans with side chains connected by a β-1-6 bond [33,34].

#### 2.1.2. Mushroom Beta-Glucans

The beta-glucan content of macrofungi depends on the species, environment, and maturity of the fruiting body, ranging between 3.1% and 46.5% [25,35,36].

Mushroom polysaccharides contain various types of glycosidic bonds and, thus, are grouped as beta-glucans, alpha-glucans, and heteroglycans. Beta-glucan molecules in individual species of fungi differ in the structure of the base chain and the number and type of bonds, as well as the type and number of side chain branches and structure (e.g., triple helix, single helix, or random helix) and in molecular weight [37]. Macrofungal beta-glucans are considered natural biological response modifiers (BRMs) [38]. Table 1 shows examples of the best-studied beta-glucans.

### 2.2. Structural Characteristics of Selected Beta-Glucans from Macrofungi

#### 2.2.1. Lentinan

**Figure 2:**
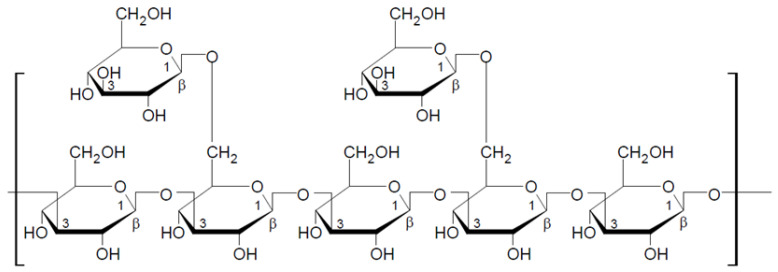
Chemical structure of lentinan [42].

Lentinan is a part of cell walls of *Lentinula edodes* (shiitake), which was first isolated in 1970 by Chihara et al. [48]. This is 1,3-1,6-β-glucan, whose basic unit is a five-molecule glucose core with two glucose side chains (one for every three glucose molecules in the basic chain) attached to the main chain by β-1,6-glycosidic bonds (Figure 2). Lentinan forms a triple helix chain in aqueous solutions. Its molecular weight varies from 300 to 800 kDa, with the average of about 500 kDa (Daltons). Lentinan primarily possesses an immune enhancement effect in tumor patients, as well as the immunomodulatory properties [49,50]. Polysaccharides extracted from *L. edodes* have not only the enhancement effect, but may also inhibit tumor growth through various mechanisms, such as inducing tumor cell apoptosis and directly killing tumor cells [51,52]. Lentinan is considered to be one of the most active components in mushrooms (*L. edodes*). It attracts many researchers due to its low toxicity and many medicinal and pharmacological properties. In addition to its immunostimulatory and anticancer properties, lentinan demonstrates antioxidant and blood lipid-lowering effects [53,54]. The latest research suggests that dietary supplementation of beta-glucans isolated from *L. edodes* may be an effective nutritional support to prevent obesity-associated cognitive decline [55].

#### 2.2.2. Schizophyllan

**Figure 3:**
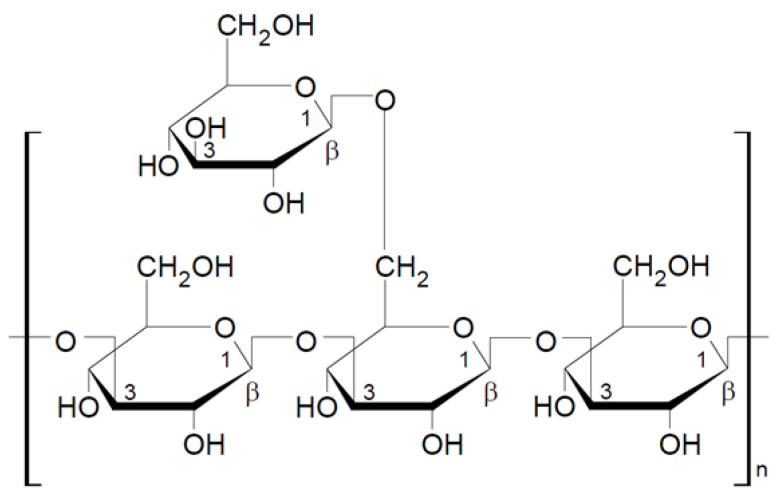
Chemical structure of schizophyllan [44].

Schizophyllan is a beta-glucan isolated from the mushroom *Schizophyllum commune*. Schizophyllan was first isolated by Kikumoto et al. in 1970 [56]. The monomeric unit of this polysaccharide consists of three glucose molecules linked by β-1-3-glycosidic bonds with one glucose side chain linked to the basic chain by a β-1-6-glycosidic bond (Figure 3). The molecular weight of schizophyllan is 100–200 kDa. As lentinan, schizophyllan forms a triple helix in aqueous solutions [44,57,58]. The properties of schizophyllan depend on several factors, including the monosaccharide composition, molecular weight, and water solubility; the extraction methods strongly influence these physicochemical properties [58].

Recent studies have shown that schizophyllan enhances the anti-inflammatory response in mouse macrophages, which may be useful in the formation of inferences during inflammatory diseases such as periodontal disease [59]. Schizophyllan has the ability to activate the dectin-1 receptor, which contributes to the increased secretion of pro-inflammatory cytokines, but at the same time strongly promotes the production of IL-10, a key anti-inflammatory cytokine that plays an important role in controlling inflammation [59].

#### 2.2.3. Krestin

**Figure 4:**
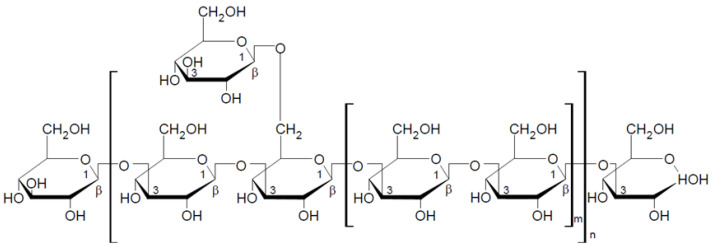
Chemical structure of the krestin repeat unit [63].

Krestin (PSK), β-glucan of arboreal fungus, is extracted from *Trametes versicolor* (L.) Lloyd. It is a protein-bound beta-glucan classified as a heteroglycan [40]. The chemical structure of the polysaccharide chain is shown in Figure 4. The molecular weight of krestin is 100,000 Da on average. Krestin, like lentinan, is a popular drug in Japan. Numerous clinical trials confirm its positive effect on the condition of patients undergoing chemotherapy due to breast, liver, stomach, colon, lung, and prostate cancer [60]. The antitumor activity of PSK lies in its ability to stimulate T lymphocytes and antigen-presenting cells, which enables proper recognition and destruction of neoplastic cells [60,61]. Krestin also shows immune-boosting and antiviral properties and hypocholesterolemic and prebiotic activity [40,62].

#### 2.2.4. Grifolan

**Figure 5:**
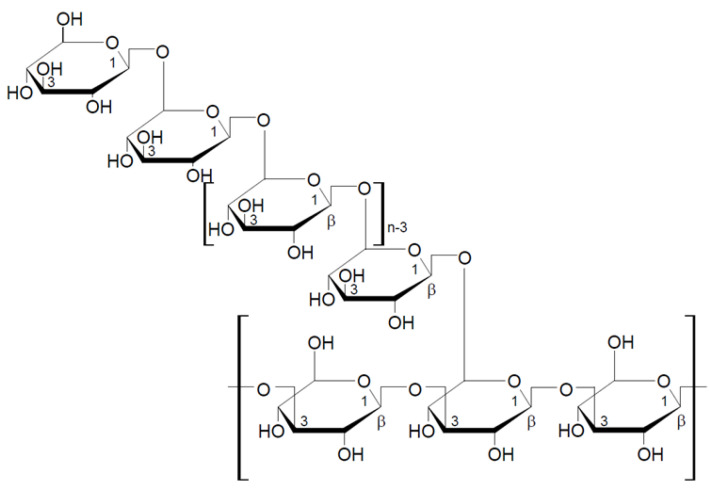
Chemical structure of the grifolan repeat unit [67].

Grifolan is a 1,3-1,6-β-D-glucan isolated from the edible mushroom *Grifola frondosa* (Dicks.) Gray. The molecular weight of this polysaccharide is about 1,000,000 Da. The monomer of grifolan molecule is built from three glucose units in the main chain and one side chain attached to the main chain by β-1,6-glycosidic bond (Figure 5). Grifolan has proven antitumor properties when administered orally, demonstrated by in vivo anti-tumor testing and in mouse tumor models [64,65]. It is one of the most effective beta-glucans that can be used in supporting the treatment of diabetes. One study found that the oral administration of submerged-culture mycelia and broth of *Grifola frondosa* improved hyperglycemia and diabetes-induced alterations in cell-mediated and innate immunities in T2DM rats [66].

#### 2.2.5. Pleuran

**Figure 6:**
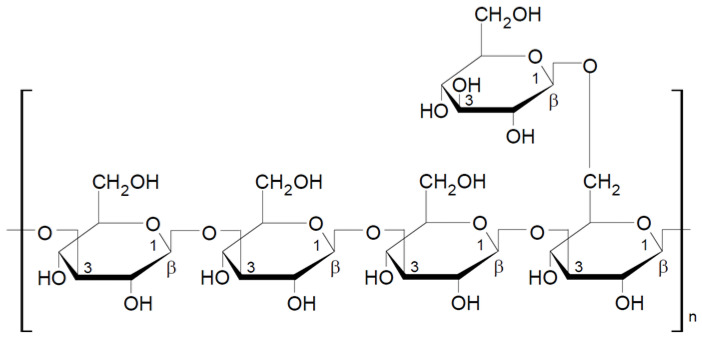
Chemical structure of the pleuran repeat unit [49].

Pleuran is 1,3-1,6-β-glucan extracted from *Pleurotus ostreatus* and sold as a dietary supplement under the commercial name Immunoglukan. The basic unit of this polysaccharide consists of four glucose molecules connected by β-1-3-glycosidic bonds, and every fourth glucose unit is linked by a side chain with a β-1-6-glycosidic bond (Figure 6). The molecular weight of pleuran is between 600,000 and 700,000 Da [49].

Pleuran’s healing properties and the ability to rebuild the epithelium have been scientifically proven in infections of the respiratory system [68,69]. It has been also found to have anti-viral properties against HSV (Herpes Simplex Virus) [70].

### 2.3. Mechanisms of Action of Beta-Glucans in the Human Body

Biological activity of fungal polysaccharides may vary depending on the type of structural monomers, the size of the molecule, the degree of its branching, and solubility in water, as well as on the structure that beta-glucans adopt in the presence of water. Studies show that high-molecular-weight molecules with β-1-3-bonds in the base chain have the best anticancer properties [71,72].

Most β-1,3-glucans show resistance to gastric juice. In an unchanged form, they pass into the small intestine, where they bind to macrophage receptors (dectin-1) in the intestinal wall and are then transported to the spleen, lymph nodes, and bone marrow. In macrophages, high-molecular-weight β-glucans are degraded into smaller fragments, which are then bound by complement receptors 3 (CR3) found on immune cells, including granulocytes. Thus, the immune response directed against tumor cells is stimulated [73].

**Figure 7:**
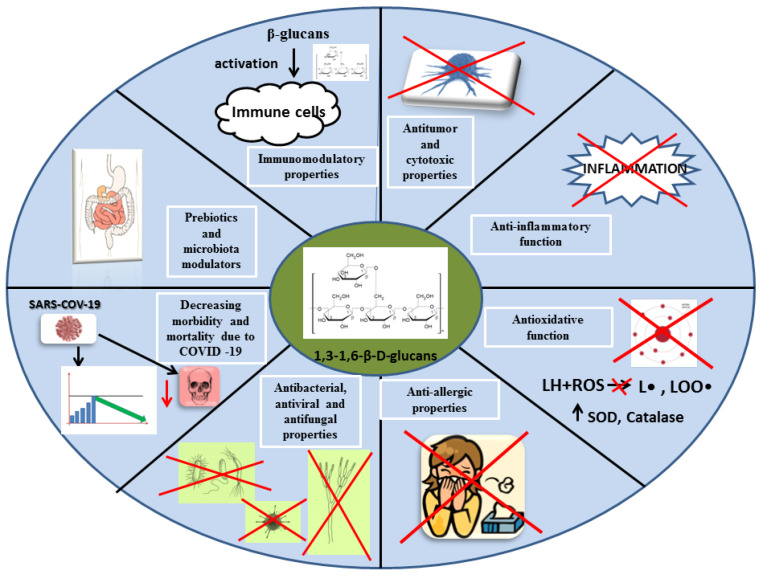
Biological activities of beta-glucans in the human body. SOD, superoxide dismutase; LH, polyunsaturated fatty acid; ROS, reactive oxygen species.; L•, alkyl radical; LOO•, superoxide radical.

A large diversity in the structure of the beta-glucan chain affects their diverse biological activity. Previous scientific reports attributed immunomodulatory, anticarcinogenic, hypolipemic, hypoglycemic, and protective effects on the circulatory system to beta-glucans. However, most of the properties of mushroom beta-glucans are due to their effects on the host immune system [74,75]. Figure 7 shows selected functions of beta-glucans in the human body.

#### 2.3.1. Immunomodulatory Properties of Beta-Glucans

Among all the mushroom-derived beta-glucans tested, most showed immunomodulatory activity. The effect on the immune system is based on the ability of beta-glucans to bind to receptors such as dectin-1, toll-like receptors (TLRs), complement receptors type 3 (CR3), scavenger receptors (Src), and lactosylceramide receptors (LacCer) present on immune cells [76].

Dectin-1 is the most abundant receptor present on dendritic cells, monocytes, macrophages, neutrophils, and T lymphocytes. The activation of dectin-1 leads to the stimulation of phagocytosis, endocytosis, and the production of reactive oxygen species (ROS) directed against pathogenic microorganisms [77]. Dectin-1 also stimulates the production of cytokines (TNF-α, IL-2, IL-10, IL-12) [78].

TLRs (toll-like receptors) are very important receptors of the immune system. They are essential in the early stages of infection to initiate an effective innate immune response. At a later stage of infection, they regulate the adaptive immune response [79]. Beta-glucan molecules, after binding to TLR 2 or TLR 4, activate the innate immune response [80]. The stimulated TLR 2 via nuclear factor NF-κB induces the production of cytokines, among them, TNF-α and IL-12 [81,82].

Complement Receptor Type 3 (CR3) is found mainly on neutrophils, monocytes, and NK cells (natural killers), but not on macrophages [81,83]. Attachment of β-glucans to CR3 increases leukocyte adhesion to microbial cells and activates the cytotoxicity pathway directed against tumor cells [81,84,85].

Src receptors (Scavenger receptors) are located primarily on endothelial cells [86]. Receptors stimulated by beta-glucans, e.g., lentinan, trigger the activation of a number of signaling pathways in the human immune system [87]. Among others, they are responsible for the activation of mitogen-activated kinases (MAPK), phosphatidylinositol kinase (PI3K), and endothelial nitric oxide synthase (eNOS) [88].

Lactosylceramide (LacCer) receptors are located on neutrophils and endothelial cells. The receptors on endothelial cells, stimulated by 1,3-β-glucans, contribute to the activation of NF-κB and the synthesis of macrophage inflammatory protein (MIP-2) and TNF-α [89]. Stimulated receptors on neutrophils cause increased ROS production necessary to inactivate pathogenic microorganisms through activation of the MAPK and PI3K cascades [90].

**Figure 8:**
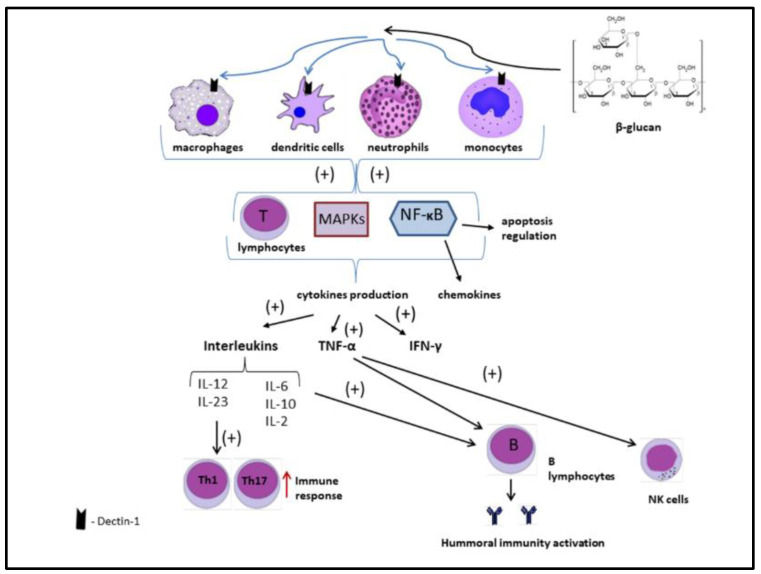
Mechanism of immunomodulatory action of beta-glucans. Authors’ elaboration based on [6,8,91].

Figure 8 shows the possible β-glucan immunomodulatory mechanism of action in the human body.

Beta-glucans bind to dectin-1 receptors located on macrophages, dendritic cells, neutrophils, and monocytes. This combination results in the activation of many compounds responsible for the immune response, including, among others, nuclear factor kappa-B (NF-κB). When NF-κB is activated, it imports to nucleus and binds specific DNA sites. In the signaling pathway, NF-κB is downstream of mitogen-activated protein kinases (MAPKs). Additionally, T lymphocytes are stimulated. Chemokines and cytokines, including interleukins, interferon-γ (IFN-γ), and tumor necrosis factor alpha (TNF-α), are released. As a result, the cellular and humoral response of the immune system is enhanced [6,8,91].

Beta-glucans derived from *Pleurotus* are attributed with the strongest immunomodulatory properties, which include stimulation of phagocytosis directed against pathogenic microorganisms [92,93].

#### 2.3.2. Antitumor and Cytotoxic Properties of Beta-Glucans

The mechanisms involved in the anticancer effects of beta-glucans are not fully understood. Until recently, it was believed that beta-glucans do not possess cytotoxic properties directed against cancer cells and do not trigger apoptotic activity [37]. So far, the described mechanisms of anti-cancer action of beta-glucans have been based on their indirect action through activity towards cells of the immune system [94]. However, scientific reports indicate the cytotoxic activity of beta-glucans isolated from *Agaricus bisporus* and the *Lactarius rufus*, directed against liver cancer cells (HepG2) [95].

Currently, the most well-known polysaccharides with anticancer activity are lentinan, schizophyllan, and krestin, which are proposed as complementary therapy for cancer treatment, especially in Japan [37,96,97,98,99].

The anticancer mechanism of beta-glucans shows synergistic effects with monoclonal antibodies used in cancer therapy [81,100]. Apart from those described above, the anticancer function of polysaccharides has been observed in many types of mushrooms, including *Agaricus*, *Ganoderma*, *Pleurotus*, and *Lentinus*. The anticancer properties of polysaccharides have been proven for colorectal [101,102,103], lung [104], gastric [37,105], and cervical cancers [106,107].

The increasing number of cancer cases contributes to the search for substances with anti-cancer effects. Bioactive substances of natural origin, which are safer and cheaper than drugs commonly used in chemotherapy, are receiving increasing attention in the scientific community. Extensive research on polysaccharides of fungal origin has been going on for several decades. Several of them have already been officially registered as drugs [108,109,110].

#### 2.3.3. Anti-Inflammatory Function of Beta-Glucans

The best-studied fungal polysaccharides with anti-inflammatory properties are heteroglycans (β-D-glucans with side chains of xylose, mannose, galactose, and glucuronic acid) [111,112,113].

Oral administration of beta-glucans isolated from fungi produced similar effects in animal models to those of non-steroidal anti-inflammatory drugs and glucocorticoids. Therefore, one of the suggested mechanisms of anti-inflammatory action of beta-glucans is inhibition of the production of pro-inflammatory cytokines (e.g., interleukin 1β) [114]. Another mechanism suggested for the anti-inflammatory properties of beta-glucans is their ability to inhibit the enzymes cyclooxygenase-2 and nitric oxide synthase [115]. The anti-inflammatory function of beta-glucans is also important in the prevention and treatment of neurodegenerative diseases such as Parkinson disease and Alzheimer disease [116]. The abovementioned polysaccharides with anti-inflammatory properties have been isolated from *Agaricus blazei* and *Lactarius rufus*, among others [113,117].

#### 2.3.4. Antioxidant Properties of Beta-Glucans

*Pleurotus* mushrooms are considered to be one of the most valuable mushrooms in terms of health. They are an excellent source of numerous bioactive compounds including polysaccharides. Beta-glucans isolated from fungi of the genus *Pleurotus* possess numerous therapeutic properties, including antioxidant effects [118,119]. Mannogalactoglucan isolated from the species *Pleurotus sajor-caju* exhibits free radical scavenging, reducing and chelating properties towards iron ions [120]. Antioxidant properties of polysaccharides of fungal origin were also observed in two polysaccharide fractions, PSPO-1a and PSPO-4a [119,121]. Polysaccharides isolated from *Pleurotus ostreatus* show strong reducing properties against the 2,2-diphenyl-1-picrylhydrazyl (DPPH) radical and the superoxide anion radical [119]. Polysaccharides isolated from *Armillaria mellea* exhibit antioxidant properties based on their ability to reduce the DPPH radical, chelate transition metals, and have strong reducing properties [122]. Polysaccharides isolated from *Trametes versicolor*, *Agaricus* spp., and *L. edodes* show significant antioxidant properties [123,124,125]. They have chelating properties that reduce lipid oxidation. Polysaccharide extracts from *G. lucidum, Ganoderma tsugae,* and *Polyporus dermoporus* have the ability to scavenge free radicals as well as to counteract a respiratory burst leading to ROS formation [126,127,128]. Polysaccharides from *Morchella esculenta* in laboratory mice showed potent antioxidant activity directed against the most potent oxidant in living organisms, the hydroxyl radical [129]. The polysaccharide significantly reduced the production of malondialdehyde (an indicator of the lipid peroxidation process) in serum and liver cells of laboratory animals [129]. 

It is worth nothing that mushroom-derived polysaccharide molecules exhibit greater antioxidant activity than monosaccharides because the polymeric chains have a greater ability to extract anomeric hydrogen and to inactivate free radicals [130].

#### 2.3.5. Beta-Glucans in the Treatment of Allergies

Currently, a large increase in allergic diseases is observed among populations worldwide. None of the civilization diseases show such a growth rate. Allergy is called an abnormal, excessive reaction of the immune system to various substances present in the environment, which we call allergens. Based on the route of entry of the allergen into the body, allergies are divided into inhalation, food, contact, venom reaction, and drug reaction [131,132].

Numerous in vitro, laboratory animal, and clinical studies indicate the anti-allergic function of beta-glucans [133,134,135]. The anti-allergic properties of beta-glucans are mainly attributed to 1,3-1,6-β-glucans found in fungi. A study involving the administration of beta-glucans to laboratory mice with asthma confirmed the healing effect of beta-glucans, which was similar to treatment with dexamethasone [136]. Moreover, one study carried out among children with recurrent respiratory infections confirmed the significant anti-allergic function of pleuran [137]. After 6 months of administration of this polysaccharide at 10 mg/kg, a significant reduction in peripheral eosinophilia and stabilization of total class E immunoglobulin (IgE) was observed [137]. In the study by Sarinho et al. [138], the administration of mushroom-derived beta-glucans to patients with asthma resulted in an increased production of the anti-inflammatory interleukin 10 (IL-10). A Japanese clinical trial involving oral administration of lentinan to allergy patients also confirmed a reduction in serum levels of immunoglobulin class E (IgE) [139].

The majority of studies conducted so far have confirmed that oral administration of polysaccharides, mainly beta-glucans isolated from *Basidiomycetes*, may prevent allergies by decreasing the level of immunoglobulin class E (IgE) and increasing the production of IFN-γ (interferon-gamma) [134,140,141].

#### 2.3.6. Antibacterial, Antiviral, and Antifungal Properties of Mushroom Beta-Glucans

There are a number of compounds in mushrooms that can inhibit the growth of microorganisms in humans. Numerous natural antibiotics and antiviral substances have been isolated from mushroom fruiting bodies, including triterpenes, ganodermadiol, ganodermic acid, and lucidol, showing activity against herpes virus, influenza, and HIV [142,143,144]. Polysaccharides, mainly β-glucans, are also responsible for their microbial inhibitory properties [145,146]. The mechanism of action of glucans against microorganisms mainly involves the activation of several different immunomodulatory mechanisms, including phagocytosis, in which the phagocytic cells of the immune system, neutrophils, and macrophages participate [134,147].

The first studies on the antimicrobial activity of beta-glucans were conducted in the 1980s using yeast β-glucans [147]. They found the protective effect of β-glucans against infections caused by *Staphylococcus aureus* [147,148]. An inhibitory effect of lentinan on the development of tuberculosis was observed through stimulation of macrophages [149]. In the animal studies, the addition of beta-glucans to the food of various fish species resulted in an increase in their resistance to pathogenic bacteria of the *Aeromonas* and *Vibrio* genera [150,151]. A number of studies described the great antiviral potential of beta-glucans [152,153,154,155]. The first experiments on the antiviral activity of β-glucans were performed on tobacco plants, and antiviral effects were observed with lentinan, schizophyllan, and zymosan [156,157]. In the 1990s, a positive treatment effect was observed in HIV patients, when lentinan was administered together with the antiretroviral drug didanosine [153]. A significant increase in the percentage of helper T lymphocytes (Th) was observed, greater than when the drug was administered alone [153]. Similar results were obtained by US researchers 16 years later [154]. Studies showed that lentinan also exhibits inhibitory effects on the replication of the herpes simplex virus (HSV), mumps, polio, measles, and viral encephalitis virus [158]. The evidence shows that β-glucans can reduce the incidence of lower respiratory tract infections and decrease the frequency of the flu-like diseases in children [68]. 

An inhibitory effect of polysaccharides isolated from *Auricularia auricula-judae* on Newcastle disease virus (NDV) was observed in Chinese studies conducted on chicken embryos [159]. Dietary lentinan supplementation maintained normal function of piglets even when they were infected with rotavirus, as reflected by reduced growth, performance loss, and diarrhea prevalence, and maintained gut immunity [160].

Currently, opportunistic microscopic fungal infections are very common due to the widely used antibiotic therapy. Candidiasis is particularly dangerous in immunocompromised patients, e.g., HIV-infected or cancer patients. It has been proven that edible mushrooms, due to the presence of numerous bioactive compounds, such as agrocybin, ganodermine, pleurostrin, or eryngin, inhibit the growth of microscopic fungi of the genera *Fusarium*, *Mycosphaerella,* and *Physalospora* [152,161,162,163]. 

Beta-glucans of macrofungi also play an important role in the protection against mycoses. The dectin-1 receptor present on immune cells plays a significant role in their activity. It has the ability to bind to certain β-glucans. Thus, β-glucans of edible mushrooms stimulate cell phagocytosis, increasing the non-specific cellular response of the host immune system directed against pathogens [164]. Another receptor, toll-like receptors (TRL) located on phagocytic cells, plays an important role in controlling fungal infections [165,166].

#### 2.3.7. Potential Role for Beta-Glucans in Decreasing Morbidity and Mortality Due to COVID-19

Since December 2019, coronavirus disease 2019 (COVID-19) caused by the severe acute respiratory syndrome corona virus 2 (SARS-CoV-2) has rapidly spread all over the world. A significant proportion of patients infected with SARS-CoV-2 develops a mildly symptomatic infection, but also a large part of patients experiences serious complications including acute respiratory distress syndrome (ARDS). ARDS is characterized by extensive inflammation of the lungs, which requires intensive care [167]. 

COVID-19 infections are characterized by pro-inflammatory status, with high levels of different cytokines, including (IL)-1β, IL-1Rα, IL-2, and IL-10. Critically ill patients requiring a stay in the intensive care unit were characterized by noticeably high concentrations of IL-2, IL-10, G-CSF, IP10, MCP1, MIP1A, TNFα, and IL-6 [168]. Uncontrolled production of proinflammatory interleukins and cytokines that cause inflammatory or cytokine storm (CS) in the lungs is induced by the binding of SARS-CoV-2 virus to the Toll-Like receptors (TLR) [169,170]. A high increase in proinflammatory factors such as IL-6, IL-8, IL-1β, and GM-CSF and chemokines such as CCl2, CCL-5, IP-10, and CCL3, along with reactive oxygen species in patients with COVID-19, is closely correlated with ARDS, leading to pulmonary fibrosis and death [168]. All changes in cytokine levels are related to various changes in cellular ingredients of the immune response, which shows close association between infection with COVID-19 and individual response from the immune system, resulting in different clinical symptoms [170].

Zhang et al. showed that anti-inflammatory therapy (suppression of pro-inflammatory interleukins, such as IL-1 and IL-6) can have a therapeutic effect in inflammatory diseases including viral infections [167]. This study found that the course of infection caused by the SARS-CoV-2 virus largely depends on the functioning of the individual immune system [167]. The innate immune system plays a crucial role in the early recognition of infecting pathogens and activation of a pro-inflammatory response, which is the first line of defense in various infections [170]. Recent studies have shown that the innate immune system may possess some form of memory called Trained Immunity (TRIM) [171]. Cells of the innate immune system stimulated with some factors, e.g., BCG vaccine (Bacillus Calmette–Guérin vaccine) or beta-glucans, go through metabolic, mitochondrial, and epigenetic reprogramming, with an outcome in a memory phenotype of an enhanced immune responses [172]. Beta-glucans can stimulate the immune responses and can act as a training agent, which leads to increased immune response when these trained cells are exposed to a secondary stimulus in the form of pathogens [172]. It was shown that β-glucans used as the training factor demonstrate protective activity against secondary fungal, bacterial, or viral infections [173,174]. 

**Figure 9:**
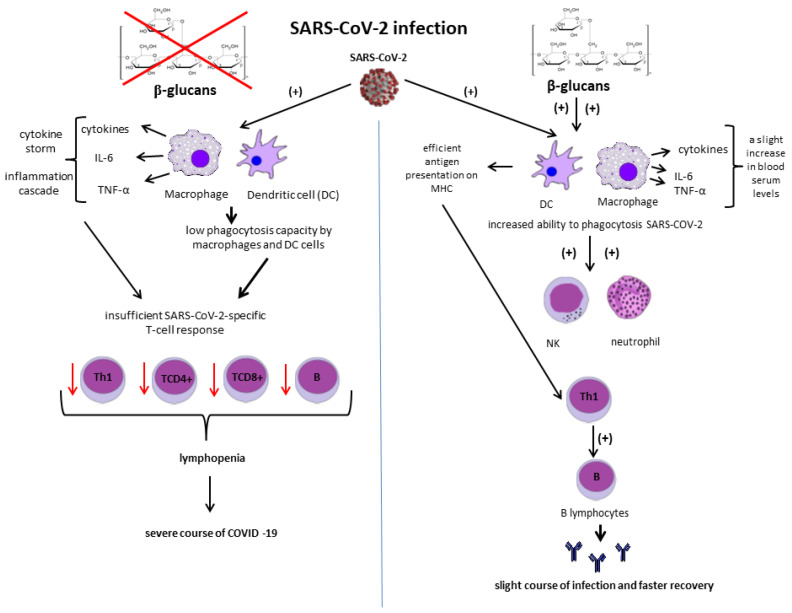
Possible mechanism of action of beta-glucans during SARS-CoV-2 infection. Authors’ elaboration based on [172,175].

Figure 9 shows a possible mechanism of action of beta-glucans during infection with SARS-CoV-2.

β-glucans activate macrophages and DC (Dendritic Cells) via appropriate receptors (Dectin-1, TLR, CR3). This results in an enhanced ability to phagocytose and efficiently present antigen on the MHC. Furthermore, β-glucan-induced macrophages can induce an enhanced defensive response of neutrophils and NK cells. Beta-glucan-induced DC cells present the virus more efficiently to T lymphocytes, which promotes stimulation of B lymphocytes and antibody production. Thus, beta-glucans may favorably influence the development of a long-term, specific, adaptive response to SARS-CoV-2 [172,175].

Geller and Yan [172] hypothesized that the use of oral administration of beta-glucan in a prophylactic setting could be an effective way to boost immune response and abrogate symptoms of COVID-19. Beta-glucans as TRIM inducers probably cause increased phagocytic capacity of macrophages and dendritic cells, which results in better processing and presentation of viral units to MHCs [84]. The 1,3-1,6-beta-glucans are considered to be the best biological response modifiers and have immunogenic properties [176]. Most glucans with this chain structure are derived from macrofungi (mushrooms) or yeast [172,177]. The hypothesis posed by Galler and Yan [172] was corroborated by other authors, who found that β-glucans from mushrooms demonstrated the potential for the treatment of lung injury [178]. In an in vitro study, *L. edodes* was shown to have potential for the treatment of COVID-19 due to its content of beta-glucans, which, through its effect on the immune system, reduces cytokine storm and, thus, ARDS [14]. This study demonstrated reduced inflammation in a lung epithelial model depending on the dose [14]. 

Another beneficial implication of the use of beta-glucans among people suffering from COVID-19 may be a decrease in the systolic and diastolic blood pressure [172]. In recent years, several studies have been published on the use of glucans in the prevention and treatment of viral diseases, especially in the context of COVID-19 (Table 2). Because of the few scientific reports to date on the function of mushrooms, beta-glucans in COVID-19 disease studies on both macrofungi and yeast are included in Table 2 for comparison purposes, as well as to highlight the high therapeutic potential of total fungal glucans in the prevention and treatment of COVID-19.

The promising results of studies on mushroom β-glucan from *Agaricus bisporus* and *Lentinula edodes* [14,175] allow us to assume a beneficial effect of these compounds in the prevention, course, and counteracting complications of COVID-19 in the era of the pandemic caused by SARS-CoV-2. Still, there are only a few studies on the promising function of mushroom β-glucans in the context of COVID-19; such research needs to be continued due to the easy availability and significant amounts of beta-glucans in mushrooms [35], as well as the safety and ease of administration without significant side effects, even in the case of insufficient purification [14,186,187]. The advantage of mushroom glucans is that they can be administered orally and have an extremely high safety profile [172].

#### 2.3.8. Mushroom Beta-Glucans as Prebiotics and Microbiota Modulators

Mushrooms owe their prebiotic properties to polysaccharides, which are not digested in the intestine. They create excellent conditions for the growth and activity of beneficial bacteria of the digestive tract of the genera *Bifidobacterium* and *Lactobacillus*. These polysaccharides include β-glucans, chitin, hemicelluloses, mannans, and xylans [192]. For example, β-glucan pullulan has proven prebiotic properties. This polysaccharide administered to the test subjects induced the development of the beneficial bacterial microflora *Bifidobacterium* [193].

Beta-glucans are resistant to human digestive enzymes and pass through the digestive tract into the intestines, retaining their original structure. Therefore, most mushrooms abundant with beta-glucans can be considered potential sources of prebiotics. Evidence for the prebiotic properties of mushroom polysaccharides is provided in the study by Synytsya et al. [21], who observed that extracts of cultivated mushrooms from *Pleurotus* genus intensively stimulated the growth of probiotic flora. In their latest in vitro study, Mitsou et al. [194] found that mushrooms rich in β-glucans may exert beneficial effects in gut microbiota and are crucial in the production of short chain fatty acids (SCFAs). Other authors emphasize that yeast glucans and mushroom glucan polymer complexes are able to stimulate the growth and development of *Lactobacillus acidophilus* and *Bifidobacterium bifidum* [195,196,197]. In their study, Mitsou et al. [194] found that all tested fungi had a positive effect on increased propionate and butyrate production. This indicates the potential of edible mushrooms rich in β-glucans as prebiotics. In addition, fungi from the genera *Pleurotus* and *Cyclocybe* presented beneficial effects on microbiota composition through the growth of *Bifidobacterium* spp. and populations of *Faecalibacterium prausnitzii* [194]. Furthermore, the available scientific evidence has shown that non-starch polysaccharides (NSPs) from various products such as oat bran, mushroom, seaweed, pectin, etc. exhibit a protective action in the treatment and prevention of inflammatory bowel disease (IBD) [198]. A decreased *Bifidobacerium*/*Faecalibacterium* (B/F) ratio is associated with obesity and type 2 diabetes [199]. Edible mushrooms can increase this ratio and maintain the microbial balance in the gut altered by a high-fat diet. Extracts of *Ganoderma lucidum* and *Antrodia cinnamomea*, polysaccharides from *Sarcodon aspratus*, have been shown to increase the B/F ratio in mice fed a high-fat diet [200,201,202].

Beta-glucans derived from fungi, due to their diversity of structures and physicochemical properties, can contribute to the growth of specific groups of bacteria that are important for human health. Unlike beta-glucans from other sources, both soluble and insoluble mushroom-derived beta-glucans support the growth of probiotic bacteria that are beneficial to consumer health [203,204].

## 3. Conclusions

β-glucans are natural molecules that have great therapeutic potential due to their immunomodulatory, antineoplastic, anti-inflammatory, antioxidant, anti-allergic, antibacterial, antifungal, and antiviral properties. Recent reports have indicated great potential for the use of beta-glucans from fungi in the prevention and treatment of COVID-19.

Beta-glucan molecules are characterized by a large diversity not only due to the source of origin (cereals, mushrooms, yeast), but also within a single species of fungus or fruiting body. Their properties can also be modified by different extraction and purification conditions. Therefore, it is very important to develop a standardized method for extraction and purification of beta-glucans and evaluation of their structure (number and length of branching and presence of amino acids, proteins, or other substituents), in order to accurately assess their mechanisms of action and potential therapeutic properties.

Previous studies indicated the potential of β-glucans in the prevention, treatment, and complications of COVID-19 [14,169,170,179,180,181,182,183,184,185,186,187,188,189,190,191,192]. Immunomodulatory, antioxidant, neuroprotective, and antithrombotic activities are of particular interest here. There are still few studies on the use of β-glucans from edible mushrooms for COVID-19 [14,175]. Edible macrofungi appear to be an excellent source of β-glucans for clinical applications, due in part to the lack of toxicological risk from fungal toxins. Because of this, edible mushrooms can be used to produce both highly purified β-glucan preparations as well as less purified cocktails. However, careful studies are needed to determine the desired formulation, to determine dosages, and to determine the feasibility of their use at different stages of COVID-19 disease.

Such data are essential to adequately support the immune system and counter COVID-19 complications while not harming it. The growing interest in the role of β-glucans in the prevention and treatment of COVID-19 may translate favorably into the development of an effective formulation for the prevention and treatment of other viruses that humanity will face in the future. Therefore, further studies on fungal β-glucans in terms of efficient extraction, purification, their activity, and mechanisms of action are needed for their most appropriate application.

## Figures and Tables

**Table 1:** Examples of studied beta-glucans of macrofungi.

Name of β-Glucan	Abbreviation	Mushroom Species	Glucan Structure	Reference
Krestin	PSK PSP	*Trametes versicolor*	1,3-β-glucan 1,4-β-glucan multi-sugar–protein complex containing mainly 1,3-β-D-glucans	[39,40]
Tylopilan	-	*Tylopillus felleus* (Bull.)	1,3-1,6-β-glucan	[41]
Lentinan	LNT	*Lentinula edodes*	1,3-1,6-β-glucan	[42]
Pleuran	-	*Pleurotus ostreatus*	1,3-β- glucan with galactose and mannose	[43]
Schizophyllan	SPG	*Schizophyllum commune*	1,3-1,6-β-glucan	[44]
MD-fraction	PDF	*Grifola frondosa*	1,6-1,3-β-glucan	[45]
Grifolan	GRN		1,3-1,6-β-glucan	[46]
Scleroglucan	SSG	*Sclerotium glucanicum* *Sclerotium rolfsii*	1,3-1,6-β-glucan	[47]

**Table 2:** Potential of yeast and mushroom glucans for the prevention, course, and complications of COVID-19.

No.	Type of Glucan	Mechanism of Action in Prevention and Treatment of COVID-19	References
1.	AFO-202-β–glucan (β-1,3-1,6 glucan from black yeast *Aureobasidium pullulans*)	Potential as a vaccine adjuvant against COVID-19; Prevention of COVID-19-associated coagulopathy.	[179]
2.	AFO-202 -β-glucans (β-1,3-1,6 glucan from black yeast *Aureobasidium pullulans*)	Regulation of blood glucose and lipid levels by β-Glucans as an indispensable tool of defense against COVID-19.	[180,181]
3.	AFO-202-β–glucan (β-1,3-1,6 glucan from black yeast *Aureobasidium pullulans*)	Immune enhancement by decreasing hyper-inflammation factors (IL-6) and minimizing the likelihood of a cytokine storm; increasing IFN-γ, sFAS, and factors like IL-7; and enhancing anti-viral cytotoxic immunity, (T cells, NK cells, macrophages, antibody production by B cells).	[168]
4.	AFO-202-β–glucan (β-1,3-1,6 glucan from black yeast *Aureobasidium pullulans*)	Immune regulatory and enhancing immune system; Immune stimulator that can activate macrophages and have positive immune actions on B-lymphocytes, natural killer cells, and suppressor T cells in the immune system.	[168,182]
5.	1,3-β-D-glucan (from *Saccharomyces cerevisiae*)	Prevention and treatment of excessive microglia activation during chronic inflammation characteristic of COVID-19 course.	[183,184]
6.	1,3-β-D-glucan (curdlan and fragmentated zymozan- proteoglucan from *Saccharomyces cerevisiae*)	Potentials to enhance microglial function and regeneration of CNS axons in COVID-19 neurological sequalae.	[184,185]
7.	1,3-1,6-β-D glucans (from shiitake mushroom *Lentinula edodes*)	Immunomodulatory and pulmonary cytoprotective effects.	[14]
8.	β-glucans (from mushrooms as *Lentinula edodes* and *Pleurotus ostreatus*)	Immunomodulating effects.	[170]
9.	β-glucan (from white button mushroom *Agaricus bisporus*)	Interrupts AR (androgen receptor)-mediated TMPRSS2 (Transmembrane protease serine 2) expression that is involved in viral entry, through its AR antagonistic activity; Attenuates serum pro-inflammatory cytokines and reduces MDSC (myeloid-derived suppressor cells) counts that are involved in the host response to viral infection, through its immunoregulatory activity).	[175]
10.	1,4-α–glucan (from *Lentinula edodes*)	Modulation and activation of NK-cells, T-cells, and γδ-T (gamma delta T).	[186]
11.	β-glucans (from edible and medicinal mushrooms)	Support the immune system before, during, and after COVID-19.	[187]
12.	Aminated β-glucan (AβG)	Potential vaccine adjuvant, immunopotentiator for simulation of antigen-presenting cells for T cells’ activation.	[188,189,190]
13.	β-glucan (from yeast, *Saccharomysces cerevisiae*)	Decreasing platelet activation by increasing TGF-β1 production. Decreasing the concentration of pro-inflammatory cytokines IL-6, which indirectly activates platelets and thrombin production. Prevention of thrombosis during the course of COVID-19.	[169,177,191]

## Data Availability

Not applicable.
